# Antimicrobial stewardship in acute exacerbations of COPD: a qualitative study of hospital pharmacists’ perspectives

**DOI:** 10.1007/s11096-026-02217-w

**Published:** 2026-09-15

**Authors:** Abdulaziz Alrashdi, Jan-Willem C. Alffenaar, Jin-Gun Cho, Bandana Saini

**Affiliations:** 1 Faculty of Medicine and Health, Sydney Pharmacy School, The University of Sydney, Camperdown, Sydney, NSW Australia; 2 Department of Pharmacology and Toxicology, College of Pharmacy, Qassim University, Al Qassim, Saudi Arabia; 3 The University of Sydney Institute for Infectious Diseases, Sydney, NSW Australia; 4 Westmead Hospital, Sydney, NSW Australia; 5 Faculty of Medicine and Health, Sydney Medical School, The University of Sydney, Sydney, NSW Australia; 6 Department of Respiratory and Sleep Medicine, Westmead Hospital, Westmead, NSW Australia; 7 The Woolcock Institute of Medical Research, Macquarie University, Sydney, NSW Australia

**Keywords:** Antimicrobial stewardship, Chronic obstructive, Pharmacists, Pulmonary disease

## Abstract

**Introduction:**

Acute exacerbations of chronic obstructive pulmonary disease (COPD) are a common cause of antibiotic use and hospital admissions. Although antimicrobial stewardship (AMS) programs are well established, the specific contribution of pharmacists to COPD-related antimicrobial decision-making remains unclear.

**Aim:**

To explore hospital pharmacists’ perspectives on their roles, responsibilities, and application of AMS principles in the management of hospitalised patients with COPD exacerbations.

**Method:**

We conducted a qualitative study using semi-structured interviews with hospital pharmacists involved in COPD care and/or AMS activities at two public tertiary hospitals in Sydney, Australia. Our research was informed by a constructivist epistemology and critical realist orientation. Interviews were conducted via Zoom, audio-recorded, transcribed verbatim, and analysed inductively using reflexive thematic analysis. Recruitment continued until data saturation was reached.

**Results:**

Seventeen pharmacists participated and three themes were identified. First, pharmacists described AMS as being a core component of their professional role, including reviewing antimicrobial indication, dose, duration, and spectrum, and promoting appropriate antimicrobial use. However, their involvement typically occurred after initial prescribing, with greater influence on treatment duration than agent selection. Second, organisational and structural factors shaped their scope of practice, including workload pressures, hierarchical prescribing cultures, limited prescribing authority, and electronic system challenges, particularly during transitions of care. Third, pharmacists described patient-centred roles alongside stewardship, including counselling on antibiotic use and discharge medicines, as well as support for inhaler technique, vaccination, and smoking cessation.

**Conclusion:**

Hospital pharmacists view AMS as a core part of managing COPD exacerbations. Their contributions include antimicrobial review, interdisciplinary communication, and patient education, but their involvement is often advisory and constrained by organisational structures, workload, and prescribing hierarchies.

**Supplementary Information:**

The online version contains supplementary material available at https://doi.org/10.1007/s11096-026-02217-w.

## Impact statements

- Hospital pharmacists are strategically positioned to drive AMS for acute exacerbations of COPD (AECOPD), yet their clinical expertise remains an underutilised resource.
- Integrating hospital pharmacists into early antimicrobial decision-making may strengthen stewardship in hospitalised patients with AECOPD.
- Hospital pharmacists’ involvement in transition of care, and preventive care may support safer antimicrobial use and continuity of COPD care.

## Introduction

Antimicrobial stewardship (AMS) has become a central strategy in addressing inappropriate antimicrobial use in hospital care, with hospital pharmacists positioned as key contributors to AMS implementation [1–3]. Evidence from hospital-based AMS programmes consistently demonstrates that pharmacist-led interventions can improve adherence to guidelines and reduce antimicrobial exposure without compromising patient safety [4–7]. However, while this evidence substantiates the effectiveness of pharmacist involvement at a programmatic level, it largely reflects measurable outcomes (e.g., utilisation, cost, duration) and offers limited insight into the underlying decision-making processes through which pharmacists interpret, negotiate, and enact stewardship in complex clinical environments. As such, the mechanisms by which pharmacists influence antimicrobial decision-making in context-specific settings remain insufficiently understood.

This limitation is particularly noticeable in the management of acute exacerbations of chronic obstructive pulmonary disease (AECOPD). COPD is a progressive disease characterised by repeated worsening of symptoms, referred to as exacerbations, which may be consequent to a variety of exposures. These exacerbations lead to high antimicrobial use, which often occurs in the context of substantial diagnostic and therapeutic uncertainty [8–11]. AECOPD is a leading cause of hospitalisation of patients with COPD globally and contributes significantly to morbidity, mortality, and healthcare utilisation [12, 13]. Despite the frequency of antimicrobial prescribing in this population, the clinical rationale for antibiotic use is often ambiguous. Exacerbations may arise from bacterial, viral, or non-infectious causes with viral pathogens frequently implicated [14–16]. Consequently, the benefits of antimicrobial therapy are heterogeneous and, in some cases, marginal. Although guidelines for the management of COPD such as Global Initiative for Chronic Obstructive Lung Disease (GOLD) advocate for a more selective approach, reserving antibiotics for patients with features suggestive of bacterial infection [11], evidence shows that prescribing practices often diverge from these recommendations, creating clinical dissonance [17]. As AMS experts, manoeuvring through these clinical uncertainties should be a routine role for pharmacists, particularly given their expanded scope of practice. Indeed, in other specialised contexts such as paediatric medicines use, hospital pharmacists are routinely consulted and are involved across drug selection and dosage adjustment, through to treatment effect monitoring [18].

Multinational qualitative research spanning over 60 countries highlights the need for effective antimicrobial stewardship (AMS) strategies tailored to chronic respiratory disease care, particularly in both acute and outpatient settings [19]. However, despite the recognised importance of AMS and the high prevalence of antimicrobial use in AECOPD, the specific role of hospital pharmacists in shaping antimicrobial decision-making in AECOPD remains poorly understood. This study therefore seeks to address this gap by exploring how hospital pharmacists understand and enact antimicrobial stewardship in the management of hospitalised patients with AECOPD. Therefore, it may contribute to a better understanding of how antimicrobial decisions are shaped and how hospital pharmacists’ roles may be strengthened to support more judicious antimicrobial use.

## Aim

The aim of this study was to explore hospital pharmacists’ perspectives on their roles, responsibilities, and application of AMS principles in the management of hospitalised patients with AECOPD.

## Method

### Study design and setting

This qualitative study used semi-structured interviews and is being reported in accordance with the Consolidated Criteria for Reporting Qualitative Research (COREQ) [20]. The study was informed by a constructivist epistemology and a critical realist ontological orientation, recognising that participants’ accounts reflect subjective experiences shaped by their clinical and organisational contexts [21].

An interview guide was developed from relevant literature and the research team’s clinical expertise (Online Appendix A). The study was conducted at two hospital sites in Sydney. The first was a tertiary referral hospital with approximately 1, 000 beds, including a high-level trauma centre and university affiliation. The second was a large tertiary acute care hospital with 534 beds. Both sites were selected due to established professional and academic affiliations with the research team.

### Participants and recruitment

Hospital pharmacists were eligible if they: (1) were registered on the National Health Practitioner Register; (2) were involved in the management of patients with COPD and/or general antimicrobial stewardship (AMS) activities; and (3) were employed at participating hospitals. Pharmacists were recruited using purposive and snowball sampling.

## Research team and reflexivity

The research team’s primary researcher was a senior hospital pharmacist (AA), a hospital pharmacy academic with expertise in antimicrobial stewardship (JWA), an experienced academic respiratory physician (JC), and an academic researcher experienced in respiratory and sleep pharmacy health services, implementation science and qualitative methods (BS). Whilst only AA maintained contact with pharmacists, participants may have known the pharmacy academics through professional circles and provided responses deemed more clinically appropriate (though their identity was maintained confidentially by AA). Similarly, the analysis may have been skewed by the academic/clinical lens rather than a pragmatic practitioner lens. During the project, ongoing fortnightly discussions were maintained within the research team. In these discussions, all researchers reflected on if, and how any researcher perspectives and assumptions may have influenced data collection, coding, and interpretation.

### Data collection

All interviews were conducted by AA using the University of Sydney Enterprise Zoom platform (Version 5.17) [22]. Audio-recordings were automatically transcribed verbatim using Zoom’s closed-captioning feature in accordance with the approved ethics protocol. Video recordings were not retained. Each participant was assigned a study code to de-identify personal data. Transcripts were cleaned, de-identified, and checked for accuracy by AA, then uploaded into NVivo Version 15 (Lumivero, Denver, CO, USA) for analysis [23]. Data were collected between May and September 2025. Recruitment continued until data saturation was reached, defined as the point at which additional interviews yielded no substantively new concepts or codes relevant to the study aims [24, 25].

### Data analysis

Data were analysed using reflexive, inductive thematic analysis following Braun and Clarke’s framework [26]. All transcripts were reviewed in full by AA for familiarisation and accuracy. Two transcripts were initially coded independently by AA and BS to support early interpretive rigour. Coding differences were discussed through iterative review of the original transcript extracts until a shared interpretive understanding was reached. Consensus was defined as agreement that coding decisions were grounded in the data, rather than as a measure of inter-coder reliability.

A preliminary coding framework was then developed and discussed with the broader research team (JWA and JC). After coding was completed, AA and BS grouped related codes into candidate themes, which were subsequently reviewed and refined with the wider research team until agreement was reached on their coherence and relevance to the study aims. NVivo was used to support coding and theme development [23].

### Ethics approval

This study received ethical approval from the Human Research Ethics Committee at Western Sydney Local Health District (approval number: 2024/ETH01617). All participants provided written informed consent.

## Results

### Participant characteristics

Seventeen hospital pharmacists participated in the study. Participants varied in experience, with the majority in early to mid-career stages, and most had received prior AMS training (Table 1). Interviews lasted for a median of 32 min.

**Table 1:** Participant demographics

Characteristic	Category	n (%)
Age group (years)	25–34	7 (41.1)
	35–44	5 (29.4)
	45–54	2 (11.7)
	>55	3 (17.6)
Sex	Male	5 (29.4)
	Female	12(70.6)
Highest qualification	Bachelor’s degree	6 (35.2)
	Master’s degree	4 (23.5)
	Doctoral degree (PhD or equivalent)	7 (41.1)
Years of experience	<5 years	2 (11.7)
	5–10 years	6 (35.2)
	11–20 years	7 (41.1)
	>20 years	2 (11.7)
Roles	AMS pharmacists	3 (17.6)
	Respiratory pharmacists	2 (11.7)
	Intensive care pharmacists	2 (11.7)
	General clinical pharmacists	10 (58.8)
Previous AMS training	Yes	12 (70.6)

Analysis generated three interrelated themes that capture how pharmacists’ stewardship practices were shaped by their temporal positioning within care processes, their location within hierarchical systems, and their reconfiguration of stewardship into patient-facing domains.

### Theme 1: Pharmacists’ role in post-prescribing optimisation rather than initial antimicrobial decision-making

As expected, pharmacists consistently described AMS as a core component of their professional role. Participants outlined routine stewardship activities including reviewing antimicrobial indication, dose, duration, and appropriateness based on patient-specific factors such as renal function, allergies, and comorbidities. Routine processes involved daily medication chart reviews and focus on reducing patient exposure to high-risk antimicrobials. However, pharmacists emphasised that they were more involved in modifying duration of therapy and reassessing ongoing need, rather than initiating or selecting antimicrobial agents in AECOPD case admissions*.*

“With COPD… it’s duration rather than dose… or selection…” (P#02).

“If IV therapy is started empirically, I set a reminder for a 48-h review and push for IV-to-oral switch…” (P#03).

Clinical uncertainty was raised as an issue with pharmacists commenting on broad-spectrum therapy initiation as a precautionary measure in the face of diagnostic ambiguity and undertreatment risks. In these situations, pharmacists described their role as more *‘reactive’* and *‘corrective’*.

“…we often see uncertainty early in admission… Sometimes prescribers default to broad-spectrum agents ‘just in case’.” (P#17).

“If it’s appropriate to… either extend the duration, or whether, if they’ve just started, they’ll say, look, maybe see for like, 7 to 10 days, see how they will go. And if it’s still deteriorating, we can extend further” (P#3).

“In ICU, they want to keep them alive… the priorities may shift… they will not hesitate’’ (P#15).

### Theme 2: Organisational constraints in enacting AMS roles in AECOPD care

Pharmacists noted several impediments to enacting AMS roles in the context of AECOPD care provision. Firstly, hospital pharmacists described a disconnect between their clinical capability and formal authority, noting that while they were confident in applying guideline-based recommendations, hospital pharmacists lacked the autonomy to independently modify antimicrobial therapy.

“…we just continue or supply therapy already initiated.” (P#02).

“…authority to adjust duration, switch IV to oral, or narrow spectrum without ‘chasing signatures’ would make big difference.” (P#17).

To navigate these constraints, pharmacists described using effective communication strategies to influence prescribing decisions, for example, by querying the rationale for a prescription and highlighting guideline-based recommendations.

“Instead of confronting prescribers like ‘antibiotic police’… we try to ask… ‘The guidelines recommend this—can you explain your reasoning?’” (P#13).

Pharmacists also reported strong hierarchical dynamics. These were especially noticeable in their interactions with junior medical staff, who were reluctant to alter prescribing decisions without senior staff endorsement, even when these were not guideline concordant.

“Even when the evidence suggests de-escalation, junior doctors may feel uncomfortable making changes without senior endorsement.” (P#10).

Other issues that hampered AMS-based involvement in AECOPD included time pressures, staffing shortages, and limited ward coverage, particularly in respiratory units, as barriers to timely antimicrobial review and intervention. Delays in review were also reported during weekends and transitions of care, contributing to prolonged or inappropriate antimicrobial use.

“We usually cover two or three wards—about 60 to 90 patients—so time is limited.” (P#8).

“Short staffing is another issue… it limits how much time we can dedicate to AMS activities.” (P#10).

Finally, pharmacists identified gaps in COPD-specific AMS training and expressed a need for more targeted education and institutional support to enhance their practice.

“Additional AMS training specific to COPD would also help.” (P#10).

### Theme 3: Roles alongside antimicrobial optimisation: patient education and transition of care

Given their limited involvement in initial antimicrobial prescribing decisions, pharmacists described contributing to AMS beyond prescription review. Their roles included patient education, counselling, smoking cessation support, inhaler technique review, and transition-of-care.

“At discharge, I review antibiotics and kidney function before sending the script to dispensary or counselling the patient myself.” (P#3).

Within these roles, pharmacists actively counselled patients on antimicrobial use, potential adverse effects, and misconceptions surrounding antibiotics, particularly during discharge. Patient beliefs, especially the perception of antibiotics as a “quick fix”, were identified as a major driver of antimicrobial demand. Pharmacists also described challenges related to limited patient receptivity to antimicrobial resistance.

“For patients, there is still a strong belief that… antibiotics are a quick fix…” (P#17).

“Many COPD patients don’t understand resistance or the consequences of unnecessary antibiotic use.” (P#02).

“A big part of my job is teaching patients…” (P#01).

“We try to educate patients during counselling, though not everyone is receptive.” (P#16).

Beyond antimicrobials, pharmacists reported engaging in broader aspects of COPD management including education on correct inhaler technique, adherence support, vaccination advocacy, smoking cessation counselling, and referral to pulmonary rehabilitation. These activities were framed within an AMS umbrella as being essential for reducing future exacerbations and, consequently, the need for antimicrobial therapy.

“Beyond treatment, we’re involved in prevention through vaccine advocacy, smoking cessation advice…” (P#06).

Overall, our findings demonstrate that hospital pharmacists do not limit their AMS role to influencing immediate antimicrobial prescribing decisions. They also contribute to patient education, transition-of-care activities, and encouraging patients to receive respiratory vaccines.

## Discussion

This study provides specific insights into hospital pharmacists’ perspectives on AMS in the management of COPD exacerbations. Overall, pharmacists regarded AMS as an integral part of their clinical role. They routinely assessed prescribed antimicrobials, and actively supported guideline-concordant prescribing. However, pharmacists expressed some disappointment in that their skills and expertise were underutilised in initial clinical decisions regarding antimicrobial prescribing, and their scope of practice in this setting was rather limited. However, pharmacists had a holistic, patient-centred view of their role in undertaking key preventive and personalised activities such as vaccine advice, inhaler technique education, COPD action plans, smoking cessation guidance, and general patient education, all of which may help reduce future exacerbations of COPD. Limited pharmacist presence in respiratory wards, and gaps in COPD-specific AMS training were also identified. These findings provide a foundation for COPD-specific pharmacist-led AMS interventions. Such interventions may help improve clinical and economic outcomes, given the prevalence and burden of COPD [4].

Consistent with the WHO framework for AMS interventions, hospital pharmacists were primarily involved in operational stewardship activities, including audit and education [3]. However, participants reported limited involvement in system-level stewardship functions, such as policy development and strategic decision-making. Pharmacists’ contributions were largely focused on implementing and monitoring AMS initiatives rather than shaping organisational stewardship policy and strategy. Additionally, pharmacists felt they had fewer opportunities to contribute at the initial stage of antimicrobial prescribing decision-making. Pharmacists described that the clinical uncertainty about an infectious cause or infecting micro-organism drove the prescribing of broad-spectrum antimicrobials. Indeed, this phenomenon has been previously reported [27]. This phenomena has also been the subject of lively debate led by antimicrobial ethicists, which aligns with Kidder’s [28] description of a dynamic ethical dilemma (i.e., ‘individual versus the community’) as well as Beauchamp and Childress’s ethical principles of autonomy (the right of the patient and prescriber), justice (individual rights versus societal benefit), non-maleficence (no harm to the patient), and beneficence (justice as opposed to mercy) [29]. In this ethical context, broad-spectrum antimicrobials may sometimes be appropriate. However, consultation with expert AMS pharmacists may help optimise antimicrobial selection.

In support of this approach, a systematic review indicated that community pharmacists can actively and appropriately prescribe antimicrobials in respiratory ailments such as acute pharyngitis in both independent and supplementary prescribing models [30]. In addition, a review of collaborative prescribing practices of hospital pharmacists showcased fifteen different types of collaborative prescribing models across more than thirty hospitals [31]. This review highlighted that hospital pharmacists’ collaborative prescribing yielded decreased length of hospital stay and costs, although ‘respiratory’ was not one of the clinical areas addressed in this review [31]. More evidence also supports the relevance of pharmacist-led AMS interventions in respiratory conditions, including AECOPD. For example, pharmacist-led review of antimicrobial duration in AECOPD has been shown to reduce mean total antibiotic duration from 7.11 to 5.31 days without increasing hospital readmission rates [32]. Given the expanding scope of pharmacy practice in hospital settings, a key recommendation for hospitals may be to collaboratively utilise pharmacists’ medicines expertise in early prescribing decisions related to AMS, and certainly in the case of AECOPD which account for a substantial proportion of hospital presentations.

Pharmacists faced several barriers during AMS implementation, including respiratory-specific training, hierarchies and resource constraints. These barriers were not unique to pharmacists. A qualitative study found that medical and nursing staff reported similar barriers to AMS adherence when caring for patients with AECOPD [27]. Moreover, the same study also identified that hospital hierarchies, and consultation etiquette limit the collaboration with non-medical staff and act as barriers to AMS implementation [23]. This aligns with our findings and suggests that these interprofessional barriers require attention within hospital systems possibly through communication skills training for health professionals involved in AMS implementation or antimicrobial decision-making. Pharmacists in our study emphasised collaborative communication. They did not want to be viewed as the “*antibiotic police*”. Instead, pharmacists preferred to be seen by medical colleagues as allies in AMS work.

This collaborative communication approach is consistent with previous research showing that pharmacists’ contributions may be shaped by professional hierarchy, limited authority, and how physicians position pharmacists within clinical decision-making [33, 34]. In the AMS context, pharmacists may therefore frame recommendations collegially rather than authoritatively to support prescriber engagement. Another key finding was that many pharmacists reported limited COPD-specific AMS training. Given that COPD exacerbations are a common reason for hospital admission, targeted training should be developed for hospital pharmacists to support AMS decision-making in this clinical context.

Pharmacists dealt not only with AMS in an AECOPD event but had a holistic view of management with the long-term view of improving antibiotic use by preventing exacerbations. For example, pharmacists identified an important AMS role by providing patient education at discharge. This included ensuring patients had a COPD action plan, could recognise early signs of exacerbation, initiate appropriate anti-inflammatory and bronchodilator therapy when indicate, and seek timely medical attention. Pharmacists expended time and effort in ensuring patients admitted to hospital with a COPD exacerbation had the appropriate skill in using inhalers and discussed preventive strategies such as smoking cessation as well as vaccinations. In community settings, similar preventive care interventions delivered by trained community pharmacists have indeed been shown to reduce exacerbations in COPD patients, with rates falling from 21 (58%) at baseline to 9 (33%) after the intervention [35].

Pharmacists supported continuity of care by extending discharge protocols to include communication with both primary care physicians and community pharmacists which may improve post-discharge outcomes and reduce the risk of future COPD exacerbations. Discharge communication between hospital pharmacists, primary care physicians, and community pharmacists could highlight key follow-up needs, including vaccination, inhaler technique errors, medication review, and adherence support. Pharmacist-to-pharmacist interprofessional communication models have been trialled in several clinical settings to improve the quality use of medicines [36, 37] with evidence demonstrating benefits, for example, in stroke care [38]. However, similar models aimed at optimising antimicrobial use in patients with COPD need to be developed. We noted that hospital pharmacists did not discuss roles such as medication review of prescribed regimens for COPD patients following discharge to ensure their treatment was adherent to guidelines. For example, COPD guidelines recommend inhaled corticosteroids only for selected patients at high risk of exacerbations and likely to benefit, given the associated risk of pneumonia with inappropriate use [39, 40]. In contrast to previous literature highlighting the role of long-term macrolide therapy in selected patients with COPD and the associated risk of colonisation with macrolide-resistant organisms [41, 42], participants did not discuss stewardship considerations related to maintenance macrolide use. Similarly, although the provision of pre-emptive antimicrobial ‘rescue packs’ for patients with COPD has been identified as a potential source of inappropriate antibiotic use in primary care [43], this practice was not raised by participants. This absence may reflect the hospital-based focus of participants’ roles, as rescue packs are more commonly associated with COPD self-management plans and primary care or community-based management rather than routine inpatient AMS activities.

Our findings should be interpreted considering several limitations. First, the study included hospital pharmacists from only two tertiary hospitals within the same metropolitan area. Although participants represented diverse pharmacy roles and levels of experience, their perspectives may have been shaped by shared organisational structures, AMS initiatives, institutional policies, and local prescribing cultures. Consequently, the findings may not capture the full range of views held by hospital pharmacists practising in rural, private, or other healthcare settings with different AMS infrastructures, and models of care, thereby limiting the transferability of the findings. Nevertheless, qualitative research seeks to generate rich, contextually grounded understanding rather than statistical generalisability, these findings therefore have value in planning AMS interventions for delivery by pharmacists working in respiratory health in tertiary settings.

Second, purposive and snowball sampling may have introduced self-selection bias; however, pharmacists were recruited from diverse pharmacy roles to capture a broad range of perspectives. Third, social desirability bias may also have influenced responses, given that AMS practices are closely linked to professional norms and expectations. Finally, the interviewer’s background as a hospital pharmacist may have shaped data generation and interpretation, although reflexive, team-based analysis was used to maintain grounding in the data.

Future work should examine strategies to better integrate hospital pharmacists in early antimicrobial decision making. This can be achieved by increasing pharmacists’ presence in respiratory wards and emergency departments. Targeted training within multidisciplinary teams may also strengthen their COPD-AMS interventions. Empowering pharmacists at transitions of care, particularly at discharge, may assist in proper medication management while supporting safe, patient-centred COPD care. Prior research has shown that pharmacist-led discharge interventions improve the appropriateness of oral antimicrobial prescribing and reduce adverse events without compromising patient outcomes [44]. Furthermore, integrated digital health systems could be leveraged during transitions of care to support longitudinal medication review and information sharing across care settings, helping to reduce unintended antibiotic continuation and inappropriate post-discharge antimicrobial use. Empowering pharmacists to undertake medication reconciliation, discharge counselling, antimicrobial review, and communication with primary care providers and community pharmacists may improve medication management. It may also support safe, patient-centred care for people with COPD. Future evaluations of pharmacist-led antimicrobial stewardship interventions in COPD are needed to assess their impact on antimicrobial resistance and patient outcomes.

## Conclusion

In conclusion, hospital pharmacists viewed AMS as integral to COPD exacerbation care. Their influence occurred mainly after antimicrobial therapy had been initiated, through post-prescribing review, dose optimisation, duration review, reassessment, and medication safety checks. Pharmacists also contributed through patient-centred activities, including antibiotic counselling, vaccination advice, inhaler education, and transition-of-care support. These roles may support more judicious antibiotic use and help reduce future COPD exacerbations. However, pharmacists reported limited involvement in initial antimicrobial prescribing decisions and wanted greater opportunity to contribute to initial antimicrobial prescribing decisions earlier in the patient trajectory. Future studies should evaluate the effects of pharmacist-led AMS interventions in AECOPD on patient care, antimicrobial use, antimicrobial resistance, and long-term COPD management.

## Supplementary Information

Below is the link to the electronic supplementary material.

Supplementary file1 (PDF 267 kb)

## Data Availability

The datasets generated during and/or analysed during the current study are available from the corresponding author on reasonable request.
