# Supplementary material for: Antimicrobial stewardship in acute exacerbations of COPD: a qualitative study of hospital pharmacists’ perspectives
Source: Int J Clin Pharm. 2026 Sep 15;48(6):2394–402. doi: 10.1007/s11096-026-02217-w (PMC13731461; doi:10.1007/s11096-026-02217-w)
Supplement: Supplementary file 1 [file 11096_2026_2217_MOESM1_ESM.pdf]

Study Title " Exploring Roles and Perspectives of Healthcare Professionals and Consumers in Optimising Antimicrobial Use in Chronic Obstructive Pulmonary Disease"
INTERVIEW GUIDE_HOSPITAL PHARMACISTS

Introduction: Thank you for taking part in this research and for making time for this interview. The interview should take between 30-45 minutes. In general, I will be asking you about your clinical experience regarding the role of hospital pharmacists in managing antimicrobial drugs in patients with chronic obstructive pulmonary disease exacerbations. Please note that you can stop the interview at any time or withdraw your consent to participate in this research later if you feel you do not wish to take part. You may also choose to not answer any questions that you do not want to. Please feel free to also take a break for any issues you need to attend to. Also, feel free to ask any questions if you like before we start. With your consent may I start the interview and recording now?

Question 1. First, I would like to ask you about your general clinical experience in managing people who have chronic obstructive pulmonary disease exacerbations (COPD). (2-3 minutes)

➲ **Discussion points/probes**

- Identifying people with COPD in the hospital (*referral/consultation*)
- Key clinical interventions (*Med review/Inhaler use/Adherence*)
- Prevention activities -*Smoking/vaping cessation/vaccination etc.*
- Education /self-management/in-patients consultation (e.g. *COPD action plans*)

Question 2. Next, I would like to ask specifically about medication prescribed when your COPD patients have exacerbations?
(5 minutes)

➲ **Discussion points/probes**

- Frequency of cases/admission/seasonal patterns/consultations by other health professionals
- Severity of illness when patients present/referral to pharmacist.
- Presentation type –antibiotic Rx/consultation/
  - *Main types of antibiotics on Rx*
  - *If consultation, which physicians, or department frequently do?*
- Recognising and referring those experiencing exacerbations/ at which level (e.g. ED, in-patients, discharge)?

Question 3. In general, what role do you think hospital pharmacists have in the management of COPD exacerbations? (5 minutes)

➲ **Discussion points/probes**

- Educating hospitalised patients with COPD about recognising exacerbations
- Dispensing/prescribing/dosing for antibiotics – antimicrobial stewardship?
- medication review/drug-related negative outcomes
- Prescriptions adherence to antibiotic/COPD guidelines
- Referral (respiratory physician, GPs, community pharmacy)
- hospital-based transitions of care (TOC)

Question 4. Do hospital pharmacists have a specific role in the management of antimicrobial drugs in the case of COPD exacerbations? (5 minutes)

➲ **Discussion points/probes**

- Prevention/ vaccination/education-COPD action plans
- Antimicrobial prescribing/monitoring/administration/ preparation usage appropriateness (*guideline, timin0g, selection, use duration, allergy issues, adherence, disposal, local resistance pattern*)

Question 5. What is needed to support your antimicrobial stewardship role in COPD? (5 minutes)

- Training
- Scope of practice – patient record access/prescribing /switching agents
- Support and referral from clinical wards
- A local hospital blueprint on COPD exacerbation management
- Remuneration for service/role acceptance

Question 6. What are your observations about patient's and healthcare professionals' (HCPs) perspective/attitude regarding antimicrobial use? (5 minutes)

- Understanding of antimicrobial resistance (*patients*)
- Awareness of HCPs
- Worry about flare-up/services provided/vaccine hesitancy/treatment need

Question 7. Would you like to add anything that we have not covered around COPD exacerbations and antimicrobial stewardship roles of pharmacists? (2-3 minutes)

CLOSING OUT INTERVIEW (2-3 minutes):

Reminder to complete demographic section on RedCap, thanks,
